# Solid Additive-Assisted Layer-by-Layer Processing for 19% Efficiency Binary Organic Solar Cells

**DOI:** 10.1007/s40820-023-01057-x

**Published:** 2023-04-10

**Authors:** Guanyu Ding, Tianyi Chen, Mengting Wang, Xinxin Xia, Chengliang He, Xiangjun Zheng, Yaokai Li, Di Zhou, Xinhui Lu, Lijian Zuo, Zhikang Xu, Hongzheng Chen

**Affiliations:** 1 State Key Laboratory of Silicon Materials, Department of Polymer Science and Engineering, Zhejiang University, Hangzhou, 310027 People’s Republic of China; 2 Department of Physics, The Chinese University of Hong Kong, New Territories, Hong Kong, 999077 People’s Republic of China; 3 Zhejiang University-Hangzhou Global Scientific and Technological Innovation Center, Hangzhou, 310014 People’s Republic of China; 4 MOE Key Laboratory of Macromolecular Synthesis and Functionalization, Department of Polymer Science and Engineering, Zhejiang University, Hangzhou, 310027 People’s Republic of China; 5 Key Laboratory of Adsorption and Separation Materials and Technologies of Zhejiang Province, Zhejiang University, Hangzhou, 310027 People’s Republic of China

**Keywords:** Organic solar cells, Fatty acid, Solid additive, Layer-by-layer, Vertical phase separation

## Abstract

Morphology is of great significance to the performance of organic solar cells (OSCs), since appropriate morphology could not only promote the exciton dissociation, but also reduce the charge recombination. In this work, we have developed a solid additive-assisted layer-by-layer (SAA-LBL) processing to fabricate high-efficiency OSCs. By adding the solid additive of fatty acid (FA) into polymer donor PM6 solution, controllable pre-phase separation forms between PM6 and FA. This intermixed morphology facilitates the diffusion of acceptor Y6 into the donor PM6 during the LBL processing, due to the good miscibility and fast-solvation of the FA with chloroform solution dripping. Interestingly, this results in the desired morphology with refined phase-separated domain and vertical phase-separation structure to better balance the charge transport /collection and exciton dissociation. Consequently, the binary single junction OSCs based on PM6:Y6 blend reach champion power conversion efficiency (PCE) of 18.16% with SAA-LBL processing, which can be generally applicable to diverse systems, e.g., the PM6:L8-BO-based devices and thick-film devices. The efficacy of SAA-LBL is confirmed in binary OSCs based on PM6:L8-BO, where record PCEs of 19.02% and 16.44% are realized for devices with 100 and 250 nm active layers, respectively. The work provides a simple but effective way to control the morphology for high-efficiency OSCs and demonstrates the SAA-LBL processing a promising methodology for boosting the industrial manufacturing of OSCs.

**Supplementary Information:**

The online version contains supplementary material available at 10.1007/s40820-023-01057-x.

## Highlights

- A solid additive-assisted layer-by-layer (SAA-LBL) processing was developed to facilitate the inter-diffusion between polymer donor and acceptor and optimize the morphology of quasi-planar heterojunction for high-performance organic solar cells (OSCs).
- The pre-phase separation between fatty acid and polymer donor, easily tuned via controlling the cohesive energy, is critical to form the desired vertical phase-separation morphology.
- The SAA-LBL is generally applicable to various OSC systems, and enables the record efficiency of 19.02% among the binary OSCs.

**Supplementary Information:**

The online version contains supplementary material available at 10.1007/s40820-023-01057-x.

## Graphical abstract

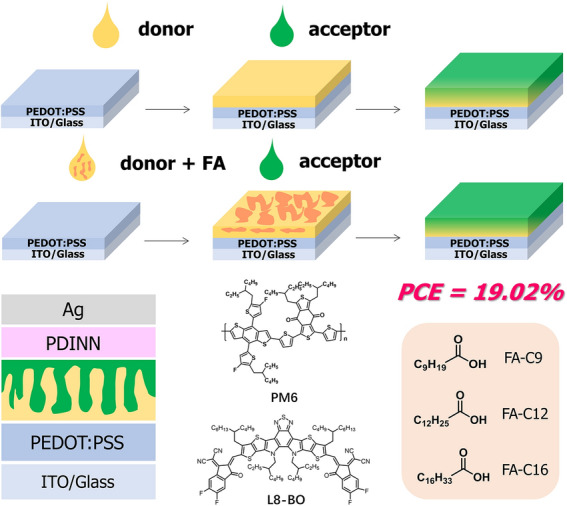

## Introduction

Organic solar cells (OSCs) have drawn worldwide attention because they are colorful, translucent, cost-effective, flexible and solution-processable for large area fabrication [1–11]. Recently, the power conversion efficiency (PCE) of single junction OSCs has exceeded 19% [12–15], which reaches the threshold for commercialization and industrial production. Besides of new molecules with better photovoltaic properties, morphology is also as important as materials in device fabrication. As is known to all, Tang et al. [16] fabricated the first organic photovoltaic device based on planar heterojunction. They evaporated two materials, donor (D) and acceptor (A), to fabricate device which is barren for exciton dissociation due to the insufficient D:A area. To solve this problem, Yu et al. [17] who developed blend casting (BC) for bulk heterojunction (BHJ) structure, blended donor and acceptor together and fabricated film with dual continuous interpenetrating network.

Till to now, the best-performed OSCs are based on the BHJ structure [18–21]. However, the morphology of BHJ is hard to control and the inappropriate morphology could lead to charge recombination for the size of phase separation is not easy to regulate. On one hand, the limited exciton diffusion length of organic semiconductor determines that there must be enough interfacial area of donor and acceptor for exciton dissociation. On the other hand, poor phase separation means reduced interfacial area, which could mitigate recombination of holes and electrons [22]. Thus, appropriate phase separation is required for charge transport and collection without affecting exciton dissociation. Unfortunately, controlling the nanostructures of donor and acceptor precisely is challenging, which limits industrial production of OSCs [23].

Nowadays, a p-i-n like quasi-planar heterojunction (QPHJ) structure fabricated with layer-by-layer (LBL) processing has been gradually considered as a more ideal structure than BHJ, for donor enriches on the top and acceptor enriches at the bottom, which could repress charge recombination and shunting between the anode and cathode [1, 24–29]. In addition, the acceptor will penetrate into the donor film and form phase separation during LBL processing. It has been demonstrated that the exciton dissociation and charge transportation will become more balanced with the LBL processing [30–33]. One of the shortcomings of the LBL processing, however, is that the diffusion of acceptor is uncontrollable and the optimal thickness of the active layer is limited by the poor inter-diffusion between donor and acceptor. Also, the morphology formed with LBL is laborious to manipulate due to the solvation and inter-diffusion entangled phase-separation process. Therefore, it is of great value to realize controllable vertical phase separation, where donor and acceptor interlace with each other uniformly and the phase separation matches the exciton diffusion length and distance of charge transport.

In this work, we report a convenient and effective strategy of solid additive-assisted LBL (SAA-LBL) processing to construct the QPHJ structure for high-efficiency OSCs. Specifically, the solid additive involves a series of fatty acids (FAs) of different cohesive energies, and the classical polymer donor PM6 and acceptor Y6 are chosen for our research. We find that by regulating the polarity and ratio of FA in PM6 film, the phase separation could be precisely tuned. During the deposition of Y6, the FA will facilitate the inter-diffusion of Y6 into PM6 film, due to their good solubility with chloroform and the Y6 molecules. The pre-formed phase separation between FA:PM6 induces more refined phase-separation structure to promote the exciton diffusion to D:A interface with a short distance. While the electrons and holes could transport to their respective electrodes through more straightforward pathways. Finally, the OSC based on PM6:Y6 via SAA-LBL processing exhibit champion PCE of 18.16%, higher than 17.52% of LBL-type devices and 16.80% of BC-type devices. With this SAA-LBL processing, the PM6:L8-BO-based devices with 95 and 250 nm films reach high PCEs of 19.02% and 16.44%, respectively, which are ones of the highest PCEs in binary OSCs with thin and thick films, validating the advantages of the SAA-LBL processing in fabrication of thick film devices. Our work demonstrates a promising approach for controlling the morphology and fabricating high efficiency OSCs.

## Experimental Methods

### Device Fabrication

**Fig. 1:**
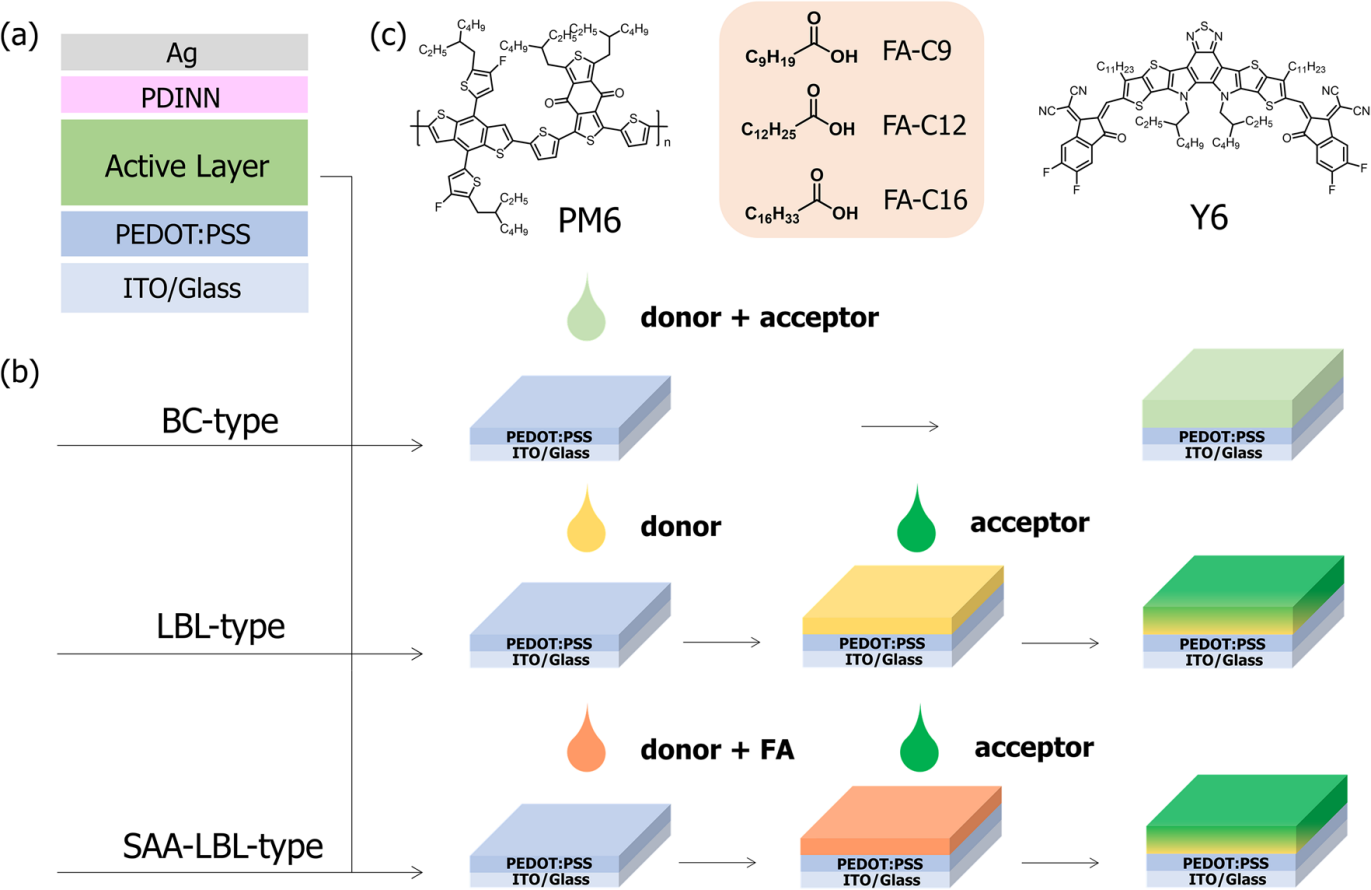
**a** Diagram of conventional device structure of OSCs. **b** Diagrams of BC, LBL and SAA-LBL processing. **c** Chemical structures of PM6, Y6, and three FAs (FA-C9, FA-C12, FA-C16)

Organic solar cells (OSCs) were fabricated on glass substrates commercially being pre-coated with a layer of indium tin oxide (ITO). The conventional structure of ITO/poly(3,4-ethylenedioxythiophene) polystyrene sulfonate (PEDOT:PSS)/Active Layer/N,N′-Bis{3-[3-(Dimethylamino)propylamino]propyl}perylene-3,4,9,10-tetracarboxylic diimide (PDINN)/Ag was adopted (Fig. 1a). Before fabrication, the substrates were cleaned with ultrasonication using detergent, deionized water, acetone, isopropanol and alcohol for 15 min in each step, and then were dried in a vacuum oven. The ITO glasses were treated in an ultraviolet ozone generator for 20 min before being spin-coated a layer of 15 nm PEDOT:PSS (Baytron P AI4083) at 4500 rpm for 30 s on ITO. Before being transferred to N_2_ glovebox, the PEDOT:PSS layer was baked in air at 150 °C for 15 min. The different approaches for active layer fabrication are shown in Fig. 1b. For BC-type, the active layer was spin coated from 16 mg/mL chloroform solution (D:A = 7:8, 0.5% v/v CN) at 2600 rpm for 25 s with thickness of around 100 nm. For BC-type devices with 10% (w/w) FAs, all of the operations are the same except the blend solution, where 10% (w/w) FAs are added. For LBL-type, the PM6 layer was first spin coated from 7 mg mL^−1^ chloroform solution at 2800 rpm for 30 s, then the Y6 layer was spin coated from 8 mg mL^−1^ chloroform solution (0.5% v/v CN) at 2500 rpm for 30 s on the top of the donor layer. The total thickness of LBL-type active layer is around 95 nm. For SAA-LBL-type, firstly the PM6 layer was spin coated from 7 mg mL^−1^ chloroform solution (with different weight ratio of FAs (0 ~ 15%) in the donor mixture) at 2800 rpm for 30 s, then the Y6 layer was spin coated from 8 mg mL^−1^ chloroform solution (0.5% v/v DIO) at 2500 rpm for 30 s on the top of the donor layer. The total thickness of SAA-LBL-type active layer is around 95 nm. For PM6:L8-BO-based devices, all conditions are the same as PM6:Y6 system except from the solvent additive is DIO with 0.25%. For thick film devices, the concentrations of PM6 and L8-BO are 12 mg/mL and 14.4 mg mL^−1^, respectively. The other fabrication details are as the same as thin film devices. All devices were treated with thermal annealing at 80 ℃ for 8 min. Then a 5 nm-thick PDINN film was spin coated as the electron transport layer from 1 mg mL^−1^ methanol solution. Finally, the Ag (100 nm) electrode was deposited by thermal evaporation to complete the device with an active area of 6.00 mm^2^.

### *J-V* and EQE Measurements

The *J-V* measurement was taken via the solar simulator (SS-X50, Enlitech) under AM 1.5G spectra whose intensity was calibrated by the certified standard silicon solar cell (KG2, SRC-2020, Enlitech) at 100 mV cm^−2^. A mask with an area of 4.73 mm^2^ (certified by National Institute of Metrology (NIM), China) was used to measure the efficiencies. The external quantum efficiency (EQE) data were gained by using the solar-cell spectral-response measurement system (RE-R, Enlitech).

## Results and Discussion

The chemical structures of PM6, Y6, and three FAs, i.e., the decanoic acid (FA-C9), tridecanoic acid (FA-C12) and heptadecanoic acid (FA-C16), are shown in Fig. 1c. The different carbon chain lengths of FAs contribute to different polarities and sequentially different miscibility with PM6, as well as different phase-separation structures.

### Photovoltaic Performances

**Fig. 2:**
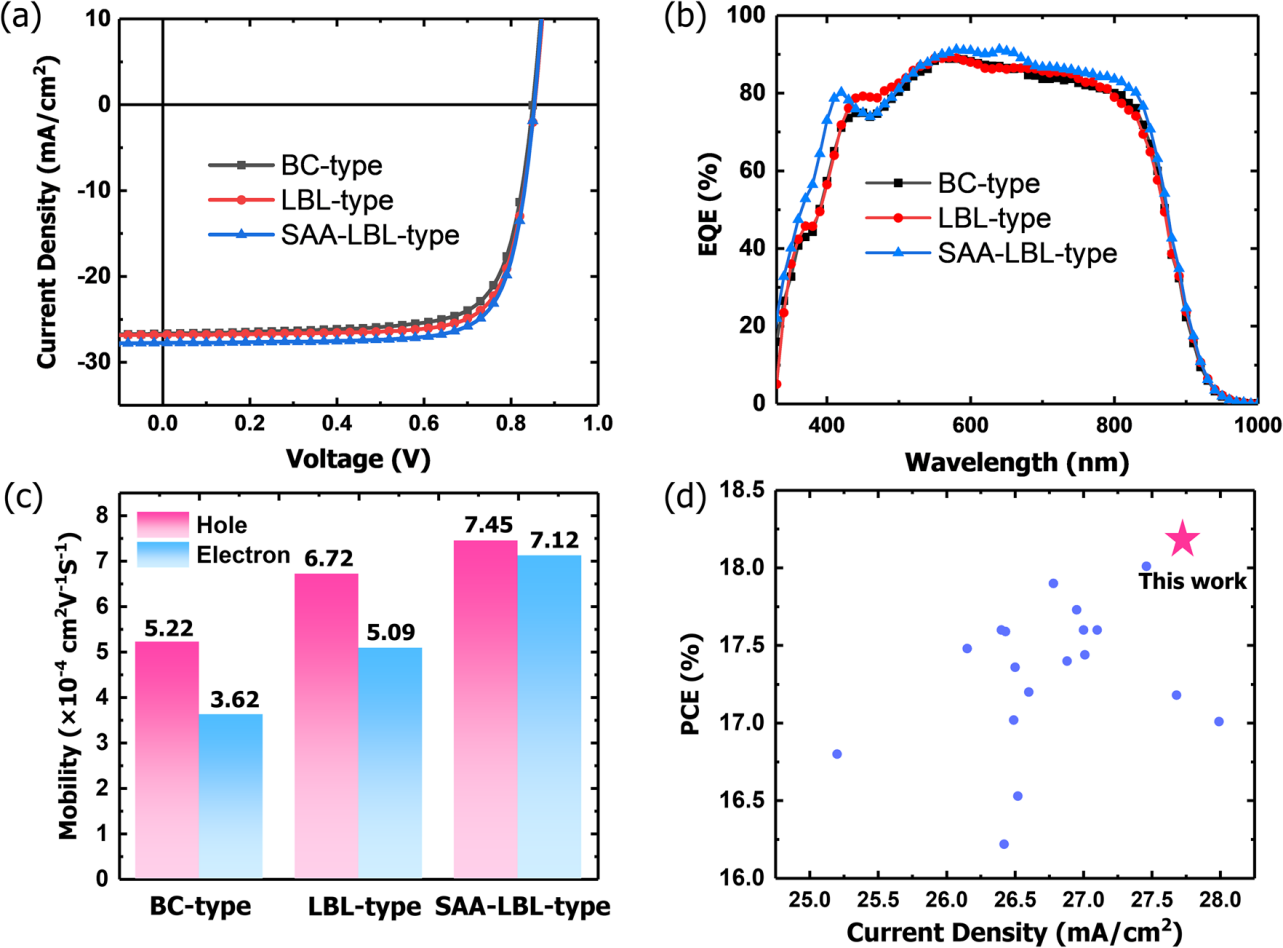
**a** *J-V* and **b** EQE curves of BC-type, LBL-type and SAA-LBL-type devices based on PM6:Y6. **c** Hole and electron mobility of BC-type, LBL-type and SAA-LBL-type devices based on PM6:Y6 tested from SCLC. **d** Plots of the PCE values versus *J*_sc_ values for the PM6:Y6-based binary OSCs reported in the literatures (see Table S1)

We first study the effect of SAA-LBL processing on the device performance of OSCs and compare it with LBL and BC processing. From the *J-V* curves presented in Fig. 2a, the BC-type device exhibits an open-circuit voltage (*V*_oc_) of 0.85 V, a short-circuit current density (*J*_sc_) of 26.66 mA cm^−2^, a fill factor (FF) of 74.1%, and a PCE of 16.80%. For LBL-type devices, a little better performance is achieved with a similar *V*_oc_ of 0.85 V, a higher *J*_sc_ of 26.81 mA cm^−2^, and an increased FF of 76.5%, and thus an improved PCE of 17.52%, which is consistent with our previous reports that LBL processing could supply not only BHJ-like interfacial area for exciton dissociation, but also D or A pure phase for charge transport and repressed charge recombination [26]. While adding 10 wt% FA-C12 into PM6 precursor donor solution, the increase in *J*_sc_ and FF occurs simultaneously, i.e., *J*_sc_ of 27.74 mA cm^−2^ and FF of 76.7%, and the PCE reaches 18.16%, which is one of the highest for PM6:Y6 devices. We measured the photostability of BC-type, LBL-type and SAA-LBL-type devices in MPP tracking under 1 sun illumination (Fig. S1). It is found that the SAA-LBL-type device exhibits slightly better photostability than the other two devices. To comprehensively compare the effect of polarity and ratio of FA on device photovoltaic performance, we investigated the effect of FA-C9, FA-C12, and FA-C16 on the device performance, as well as their various ratios. It shows that FA-C12 and FA-C16 promote the PCEs of OSCs, especially the *J*_sc_, compared to LBL-type devices without FAs, as shown in Table 1. Among the three FAs, FA-C12 presents the highest PCE. These results demonstrate that FA-C12 is an effective solid additive combined with LBL processing to cast highly efficient QPHJ devices.

**Table 1:** Photovoltaic parameters of BC-type, LBL-type and SAA-LBL-type OSCs

Active layer	Thick-ness (nm)	Solid additive^a^	Device type^b^	*V*_oc_ (V)	*J*_sc_ (mA cm^−2^)	*J*_cal_^*c*^ (mA cm^−2^)	FF (%)	PCE (%)^d^
PM6:Y6	100	/	BC-type	0.85 (0.85 ± 0.002)	26.66 (26.69 ± 0.15)	26.21	74.1 (74.2 ± 0.2)	16.80 (16.87 ± 0.13)
	95	/	LBL-type	0.85 (0.85 ± 0.002)	26.81 (26.67 ± 0.12)	26.57	76.5 (76.5 ± 0.1)	17.52 (17.46 ± 0.09)
	95	10% FA-C9		0.85 (0.85 ± 0.001)	27.12 (27.03 ± 0.14)	26.91	75.3 (75.3 ± 0.2)	17.40 (17.37 ± 0.09)
	95	10% FA-C12	SAA-LBL-type	0.85 (0.85 ± 0.002)	27.74 (27.66 ± 0.16)	27.29	76.7 (76.6 ± 0.2)	18.16 (17.93 ± 0.12)
	95	10% FA-C16		0.84 (0.84 ± 0.003)	27.17 (27.02 ± 0.12)	26.94	77.0 (76.8 ± 0.1)	17.57 (17.49 ± 0.14)
	95	5% FA-C12		0.85 (0.85 ± 0.002)	26.78 (26.65 ± 0.11)	26.36	77.3 (77.3 ± 0.1)	17.65 (17.52 ± 0.08)
	95	15% FA-C12		0.85 (0.85 ± 0.002)	26.66 (26.52 ± 0.13)	26.53	76.6 (76.6 ± 0.1)	17.36 (17.29 ± 0.06)
PM6:L8-BO	100	/	BC-type	0.88 (0.88 ± 0.003)	26.54 (26.35 ± 0.15)	26.23	79.2 (79.2 ± 0.1)	18.56 (18.50 ± 0.10)
	95	/	LBL-type	0.88 (0.88 ± 0.002)	26.76 (26.65 ± 0.14)	26.45	79.2 (79.2 ± 0.1)	18.73 (18.65 ± 0.08)
	95	10% FA-C12	SAA- LBL-type	0.88 (0.88 ± 0.002)	26.68 (26.55 ± 0.12)	26.38	80.5 (80.4 ± 0.2)	19.02 (18.92 ± 0.13)
	250	/	LBL-type	0.88 (0.88 ± 0.006)	26.82 (26.68 ± 0.17)	26.51	66.2 (66.0 ± 0.2)	15.54 (15.42 ± 0.12)
	250	10% FA-C12	SAA- LBL-type	0.88 (0.88 ± 0.007)	27.30 (27.12 ± 0.15)	26.98	69.0 (68.9 ± 0.2)	16.44 (16.33 ± 0.10)

^a^Solid additive weight ratio in donor

^b^Active area: 6.00 mm^2^, measured with a mask (area: 4.73 mm^2^, certified)

^c^Integrated current densities from EQE curves

^d^Values in the parenthesis are the average data based on ten devices

External quantum efficiency (EQE) spectra are measured to figure out the photocurrent generation, which is the major reason underlying the improved device performance in different structure devices (Fig. 2b). All three types of devices based on PM6:Y6 show high EQE peaks. The integrated current density for BC-type, LBL-type and SAA-LBL-type devices under optimal conditions is 26.21, 26.57 and 27.29 mA cm^−2^, respectively. The LBL-type devices display slightly higher EQE than BHJ devices especially in the range of 400–500 nm. The SAA-LBL-type devices show obviously higher EQE than the other two devices, specifically in the PM6 absorption range, mainly due to the optimized morphology discussed later. All the integrated current densities agree well with those gained from *J-V* curves with small deviation of < 5% (see Table 1).

To reveal the effect of different processing methods on charge recombination. The dependence of *V*_oc_ and *J*_sc_ on light intensity (*P*_light_) are measured. The slope of *V*_oc_ versus *P*_light_ is adopted to examine the type of recombination [34], as shown in Fig. S2. The slopes for BC-type, LBL-type and SAA-LBL-type devices are 1.20, 1.13 and 1.12 kT e^−1^, respectively. By fitting the *J*_sc_-*P*_light_ curves with Eq. S1, the $\alpha$ values are derived as 0.992, 0.994 and 0.996 for BC-type, LBL-type and SAA-LBL-type devices, respectively, where $\alpha$ is always used to evaluate the degree of bimolecular recombination (Fig. S2). The BC-type device has the most severe charge recombination, which agrees well with the *J-V* and EQE results. Comparing with BC-type device, the charge transport in LBL-type device is improved, as attributed to the primary vertical phase separation discussed later [35, 36]. With FA-C12, the SAA-LBL-type device exhibits the weakest charge recombination behavior. The charge transport behaviors in active layers are studied by employing space charge limited current (SCLC) measurement (Fig. S2). For BC-type device, the hole and electron mobility are calculated to be 5.22 × 10^–4^ and 3.62 × 10^–4^ cm^2^ V^−1^ s^−1^, respectively. As for LBL-type device, the hole and electron mobility increase to 6.72 × 10^–4^ and 5.09 × 10^–4^ cm^2^ V^−1^ s^−1^. With the contribution of FA-C12, mobility ascends to 7.45 × 10^–4^ and 7.12 × 10^–4^ cm^2^ V^−1^ s^−1^ for hole and electron, respectively. The results are shown in Fig. 2c. It is plausible that SAA-LBL-type performs more balanced charge transport and contributes to the higher *J*_sc_.

To examine the exciton dissociation probability (*P*_diss_) and charge collection probability (*P*_coll_) of BC-type, LBL-type and SAA-LBL-type devices, the photocurrent density (*J*_ph_) versus effective voltage (*V*_eff_) characteristics are measured (Fig. S3). Herein, the *P*_diss_ of the BC-type, LBL-type and SAA-LBL-type devices are almost the same, approaching or exceeding 0.99, implying all of them have sufficient D:A interface for exciton dissociation. However, the *P*_coll_ is different among these three heterojunctions. The SAA-LBL-type device possesses the highest one, 0.901, for its effective charge transport and reduced recombination, as a result, the FF and *J*_sc_ values get elevation simultaneously. The lowest *P*_coll_ of LBL-type devices may be caused by its monomolecular recombination due to the limit distribution of acceptor permeation (see TOF–SIMS characterization). The similar phenomenon was also observed in Peng’s research [37].

### Vertical Phase Distribution, Miscibility and Domain Size

**Fig. 3:**
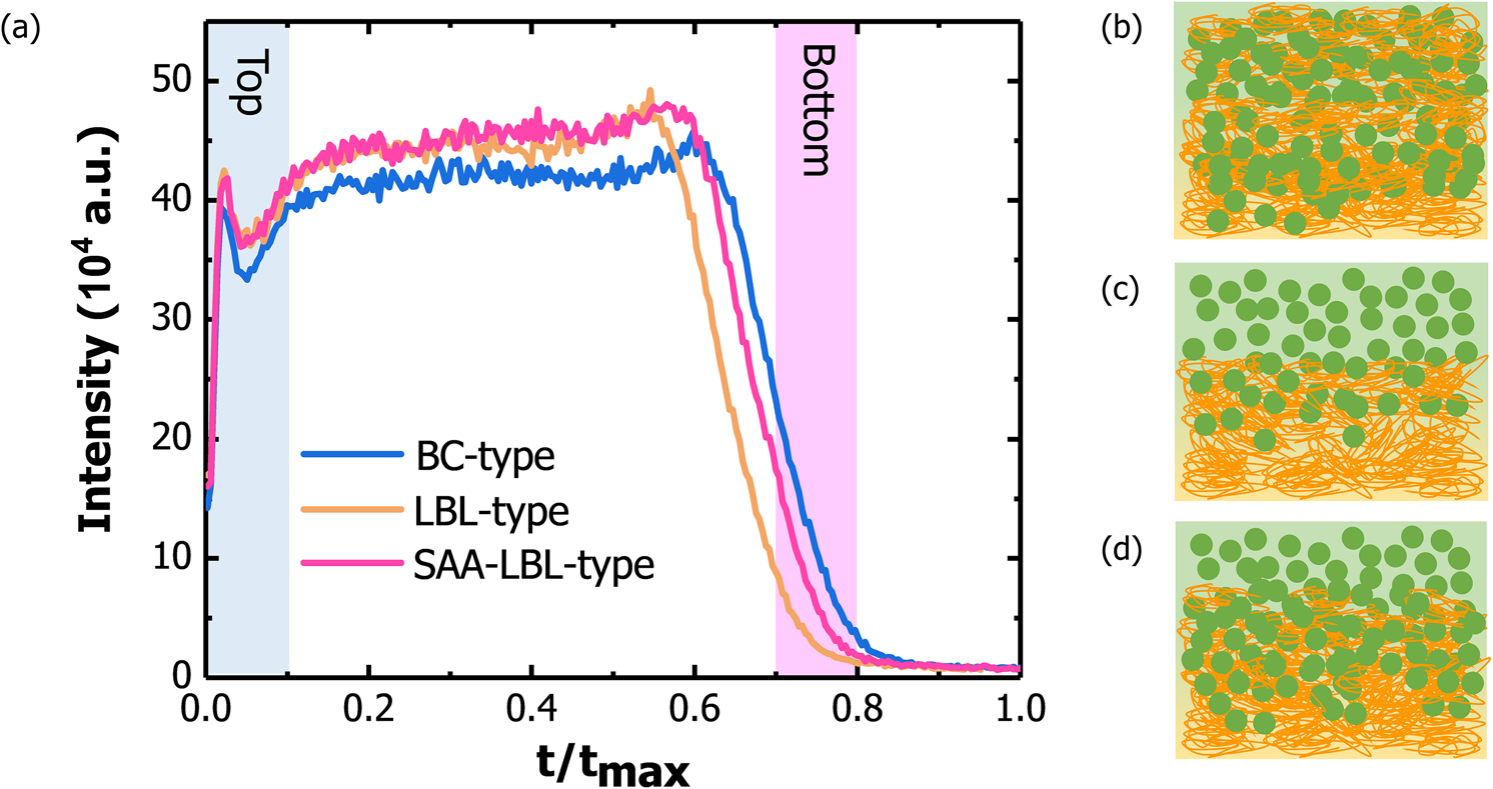
**a** TOF–SIMS ion yield of CN^−^ as a function of sputtering time for BC-type, LBL-type and SAA-LBL-type blend films. **b** Diagram of BC-type morphology. **c** Diagram of LBL-type morphology. **d** Diagram of SAA-LBL-type morphology

To demonstrate the working mechanism of FA-C12, UV–Vis, atomic force microscope (AFM), Attenuated Total Reflectance-Fourier-Transform Infrared Spectroscopy (ATR-FTIR) and time-of-flight secondary ion mass spectrometry (TOF–SIMS) are measured. From the UV–Vis spectra (Fig. S4), the addition of FA-C12 does not have obvious impact on the absorption behaviors of film, which suggests the function of acid is physical not chemical. Besides, spin-coating of pristine chloroform on PM6 layer does not affect much the uniform of the whole film (Fig. S5a, b). Though RMS of PM6 film decreases, PM6 still remains fiber network (Fig. S5c, d), which is important to charge transfer. The PM6 films containing FA display obvious carboxyl peak at around 2300 cm^−1^ in ATR-FTIR image (Fig. S6). After spin coating chloroform (CF) solution of Y6 or CF solvent, the carboxyl peak reduces to a negligible level compared to PM6, which proves that the additive FA could be removed by the sequential deposition of Y6. To verify the negligible effect of FA residual to active layer, we directly add FA-C12 into D:A blend solution to cast BC-type devices. From the results shown in Table S2, we could find even if the addition of FA up to 10% (w/w), the effect to PCE is mild. While the ATR-FTIR is an auxiliary evidence, TOF–SIMS could directly prove the vertical phase distribution of films to clarify the role of FA-C12 in device fabrication (Fig. 3a). Since CN is the exclusive group in Y6, the vertical distribution of acceptor could be traced via the vertical alteration of CN^−^ intensity. Comparing the TOF–SIMS results of three devices shown in Fig. 3a, it is found that on the top, LBL-type and SAA-LBL-type have stronger intensities of CN^−^ signal than BC-type, which means Y6 gathers more on the top. With the depth increasing, the intensities of CN^−^ signal for LBL-type and SAA-LBL-type are still stronger than BC-type, implying Y6 enriches more on the shallow layer of LBL-type and SAA-LBL-type films. At the bottom, BC-type possesses the strongest intensity of CN^−^ signal, which means more Y6 gathers at the bottom in BC-type device comparing to LBL-type and SAA-LBL-type devices. In other words, more donor gathers at the bottom in LBL-type and SAA-LBL-type than BC-type. Besides, the intensity of CN^−^ signal in SAA-LBL-type is stronger than that of LBL-type at the bottom, indicating deeper distribution of Y6 in SAA-LBL-type than LBL-type, which is beneficial for exciton dissociation and charge transport. Therefore, a desired p-i-n morphology is constructed. The diagrams of BC-type, LBL-type, SAA-LBL-type morphology are shown in Fig. 3b, c. According to our previous work, the solvent will first swell polymer donor to form a gel-like network and then the acceptor intercalates into the swollen polymer matrix to form phase separation [38]. The intermixed PM6:FA blend facilitates the solvation process and result in a deeper distribution of Y6. When spin coating Y6 solution onto pristine PM6 film, the low-boiling point CF could only swell limited PM6, but for PM6/FA-C12 blend film, FA-C12 could be easily dissolved by CF and Y6 will diffuse into the PM6 film, replacing the position of FA-C12. These results provide fundamental proof that with the assist of FA-C12, the vertical phase separation could be optimized and a more perfect p-i-n-like morphology will form to reduce charge recombination and improve charge transport.

**Fig. 4:**
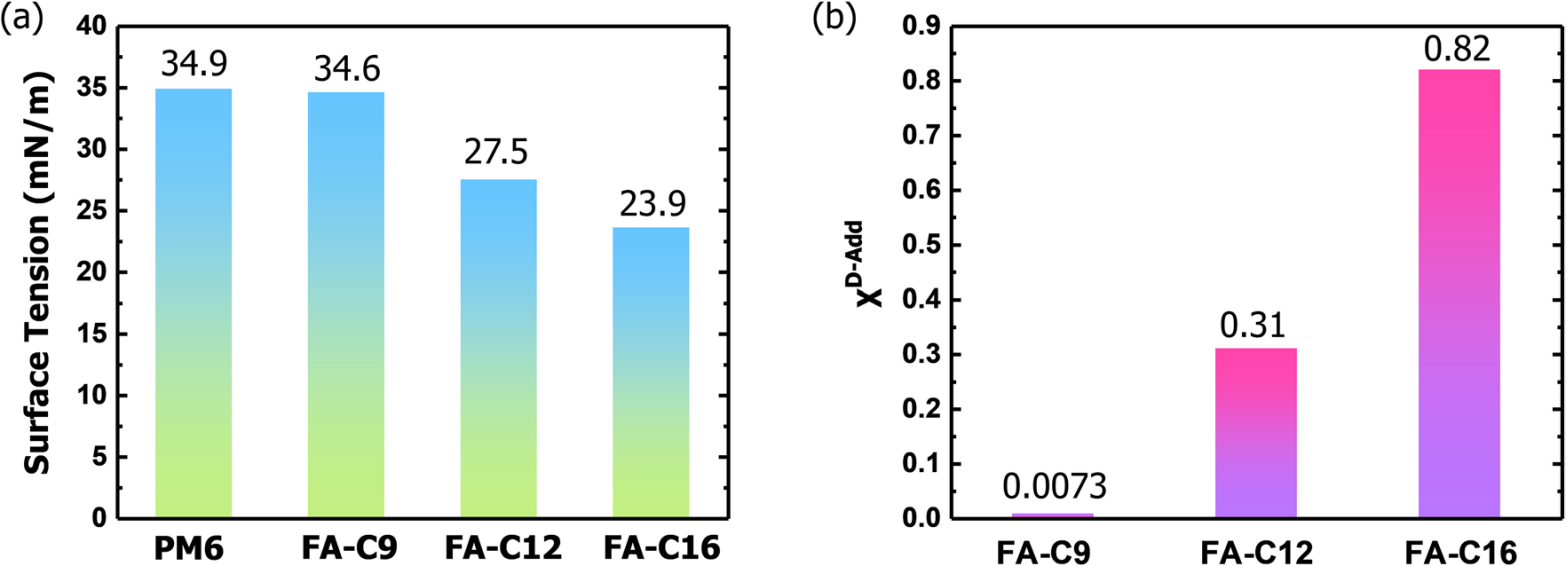
**a** Surface tension of PM6, FA-C9, FA-C12 and FA-C16. **b** *χ*^D−Add^ between PM6 and FAs

Since the polymer domain could be maintained after spin coating acceptor for the solvent CF solely swells the polymer fibril network instead of dissolving [39, 40], the FA-induced phase separation is crucial to the device morphology. We then study the reason why FA-C12 is the best. Three typical FAs with different carbon lengths, i.e., FA-C9, FA-C12 and FA-C16, are chosen. The Flory–Huggins interaction parameter *χ*^D−Add^ could be used to compare the miscibility of PM6 donor and FA additive, where small *χ*^D−Add^ means fine miscibility, otherwise, the phase separation between donor and additive will happen. Contact angle measurements are implied to calculate the *χ*^D−Add^ in the light of Owens and Wendt’s theory and Young Equation [42]. Water and diiodomethane, two solvents with different polarities, are used to measure the contact angles of different materials. The results are shown in Fig. S7. From the measurements, the surface tension of PM6, FA-C9, FA-C12 and FA-C16 are estimated to be 33.6, 34.6, 27.5 and 23.9 mN m^−1^, respectively. As a result, the *χ*^D−Add^ of PM6 with three FAs is 0.0073, 0.31 and 0.82 for FA-C9, FA-C12 and FA-C16, respectively. Figure 4 shows the comparisons and Table S2 summarizes the results. Among the three FAs, FA-C9 has the smallest value of 0.0073, indicating it could mix well with PM6, therefore the phase-separation size is small and the interfacial area of D:A will be extremely large, which leads to severe recombination of electrons and holes. Contrarily, the sufficiently large *χ*^D−Add^ of FA-C16 and PM6 contributes to incomplete phase separation, leading to slightly inadequate area for exciton dissociation. Excitingly, FA-C12 has suitable miscibility with PM6 which could not only form enough interfacial area for exciton dissociation, but also apt size of phase separation to restrain charge recombination.

The grazing incidence small angle X-ray scattering (GISAXS) are also measured to study the effect of FAs to domain size (Fig. S8). The linecut and fitting curves could be seen in Fig. S9. The domain size of BC-type film is 19 nm, and the LBL-type is 21 nm, slightly larger than BC-type due to the feature of LBL processing. For film processed with FA-C9-assisted-LBL processing, the domain size is 17 nm, which is the smallest value among these films. The domain sizes of film fabricated with FA-C12 and FA-C16-assisted LBL-processing are similar, both are 22 nm. Generally, the large domain size would facilitate to form well-connected phase domains and thus excellent charge transport pathway [41]. Combined with *χ*^D−Add^ and dependence of *V*_oc_ and *J*_sc_ on *P*_light_ (Fig. S10), it is convinced that the FA-C12 seems a suitable solid additive for LBL processing, and the morphology induced by FA-C12-assisted LBL processing seems better to reduce charge recombination and improve charge transport compared to the others. Figure S11 demonstrates the *P*_diss_ of three structures, comparing the GISAXS results, FA-C12 conducts to appropriate domain size to repress charge recombination. On the contrary, FA-C16 and FA-C9 could not provide fluent charge transport as effective as FA-C12. More than that, profited from the tunability of FAs, the polarity of FA could be easily adjusted by change the length of carbon chain and the amount of carboxyl. Hence, the miscibility of donor and FA could be well controlled so as to explore the most proper domain size.

### Morphology and Crystallinity

**Fig. 5:**
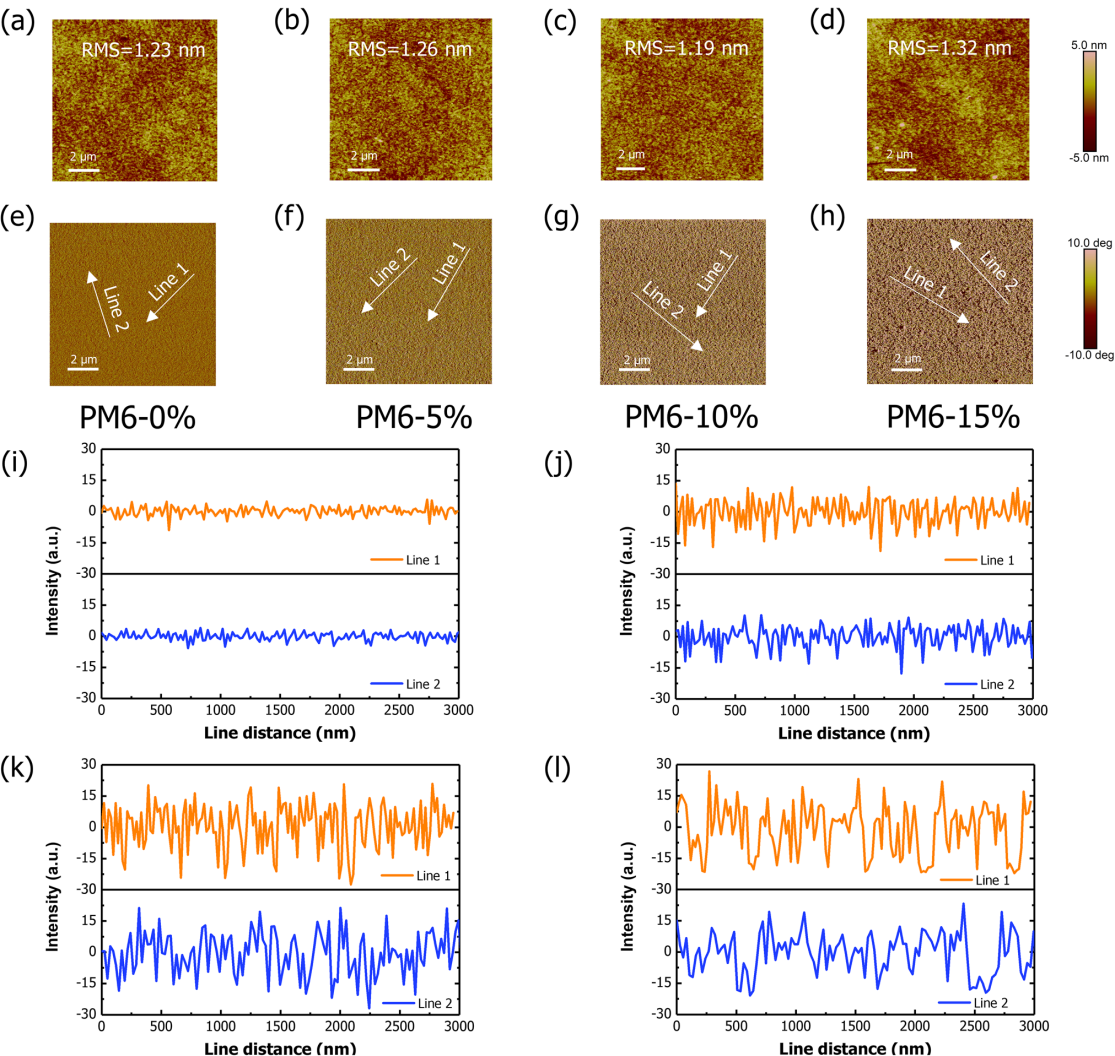
AFM height images of **a** pristine PM6 film, **b** PM6 films with 5% FA-C12, **c** PM6 film with 10% FA-C12, **d** PM6 film with 15% FA-C12. AFM phase images of **e** pristine PM6 film, **f** PM6 films with 5% FA-C12, **g** PM6 film with 10% FA-C12, **h** PM6 film with 15% FA-C12. **i** Line profiles of AFM signals of PM6 films with different ratios of FA-C12

As FA-C12 is the most suitable solid additive for device fabrication, the influence of FA-C12 ratio (0, 5, 10, 15 wt%, respectively) on the evolution of PM6 phase separation is also investigated by the AFM (Fig. 5a–h). In pristine PM6 film, the RMS is 1.23 nm, with the addition of 5% (w/w) FA-C12, the film becomes slightly rougher with RMS = 1.26 nm due to the phase separation between PM6 and FA-C12. However, the RMS decreases slightly to 1.19 nm while increasing the addition of FA-C12 to 10% (w/w). It could be seen that in the height images with the increase of FA-C12 content, the root mean square (RMS) does not have large changes but some mild differences among the four films mainly due to the plasticization of FA-C12, because phase separation will increase the roughness but plasticization is able to smooth the surface. However, the phase images offer direct proof that increased addition of FA-C12 could increase the phase separation. To make a detailed comparison, the FA-C12 phase widths of the blends with different ratios were measured. The line profiles in the AFM phase images are shown in Fig. 5i. In pristine PM6 film, the degree fluctuates around 0°, indicating even composition of film. With the increase of FA-C12, the fluctuation becomes fiercer and the distances between peaks become larger, revealing that the different ratios of FA-C12 indeed influences the phase separation and domain size of active layer. While the scale of 10 μm presents phase aggregation behaviors of PM6 and FA-C12, the scale of 1 μm reflects the fibril morphology of PM6. Figure S12 shows the fibril structures of PM6 in different addition ratios of FA-C12. It seems that with the increase of FA-C12, the fibril morphology is not disturbed. Furthermore, to understand the details of PM6 fiber, line profiles are measured. The widths of PM6 fiber shown in Fig. S12 are similar under different conditions, suggesting there is no change on the fiber morphology of PM6 with FA-C12, which also explains the excellent charge transport performance of SAA-LBL-type devices. Combined with *J-V* and EQE test (Fig. S13 and Table 1), it could be found that 10% (by wt) FA-C12 is optimal to form the appropriate phase separation and induce better photon-to-electron conversion.

**Fig. 6:**
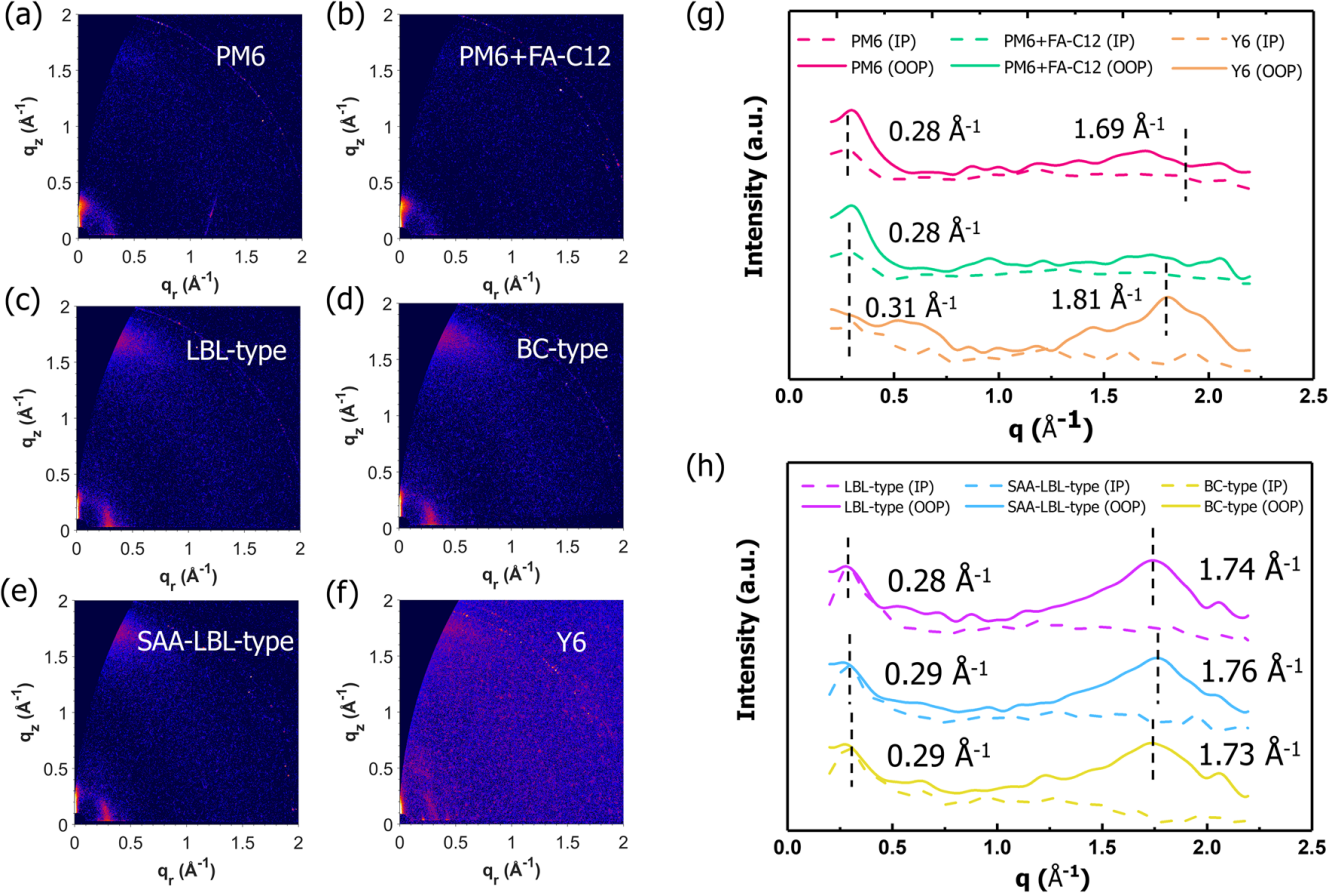
The 2D GIWAXS images of **a** PM6 pristine film, **b** PM6 film with FA-C12, **c** LBL-type thin film, **d** BC-type thin film, **e** SAA-LBL-type thin film, **f** Y6 pristine film. The 1D X-ray profiles of the corresponding **g** PM6 (w/o FA-C12) and Y6 films and **h** BC-type, LBL-type and SAA-LBL-type PM6:Y6 thin films

Grazing incidence wide angle X-ray scattering (GIWAXS) is adopted to investigate the effect of solid additive on film morphology, especially the molecular crystallinity. Figure 6a–f displays the 2D GIWAXS images of PM6 pristine film, PM6 film with FA-C12, LBL-type thin film, BC-type thin film, SAA-LBL-type thin film, and Y6 pristine film. The related data are shown in Tables S4-S7. As shown in Fig. 6g, both the pristine and PM6/FA-C12 blend film display dominant face-on orientation. The addition of FA-C12 affects the molecular packing distances and molecular packing order. In pristine PM6 film, the (100) diffraction peak locates at *q* = 0.28 Å^−1^ and (010) diffraction peak locates at *q* = 1.69 Å^−1^, so the lamellar stacking distance and π-π stacking distance could be calculated as 22.4 and 3.72 Å, respectively. For PM6/FA-C12 blend film, the lamellar distance is 22.4 Å and the π-π stacking peak almost disappear, implying the addition of FA-C12 weakens the π-π intermolecular interaction. As an impurity, FA-C12 could hinder the packing of PM6 fiber, which makes PM6 film become easier to be swollen and Y6 easier to diffuse and intercalate with donor PM6. The crystal coherent lengths (CCL) for the lamellar stacking (CCL_100_) and π-π stacking (CCL_010_) all decrease after adding FA-C12, which indicates the addition of FA-C12 will slightly reduce the lamellar stacking of PM6, but obviously affect the π-π stacking of PM6. As for blend films shown in Fig. 6h, all three structures exhibit strong face-on orientation with a π-π stacking peak observed at about *q* = 1.73 Å^−1^ in the out-of-plane (OOP) direction with π-π stacking distance of 3.63 Å, while SAA-LBL-type film has a mildly larger *q*_z_ than the others, thus closer π-π stacking. In the in-plane (IP) direction, the lamellar stacking peaks of all three structures appear at around 0.29 Å^−1^, corresponding to a lamellar distance of 21.7 Å. The CCL_100_ of BC-type, LBL-type and SAA-LBL-type are 28.4, 31.4 and 34.7 Å, respectively. The increased CCL_100_ in LBL-type and SAA-LBL-type compared to pristine film implies the CF will swell a fraction of PM6 during deposition of Y6, which makes the PM6 chains reorganize and thus contributing to the more order stacking of PM6. The larger CCL_100_ sizes of LBL-type and SAA-LBL-type than BC-type accord well with increased EQE value of PM6 range (Fig. 2b). For π-π stacking CCL, the SAA-LBL-type and LBL-type film have similar value, larger than BC-type, which indicates that LBL processing could reduce the destroy of intermolecular interactions caused by blend of donor and acceptor. Besides, the lamellar peaks originated from PM6 and π-π stacking peaks derived from Y6 in the OOP direction could all be preserved in blend films with strong intensities, manifesting the FA-C12 has no harm to the crystallinities of donor and acceptors in the process of film formation.

### Charge Transfer, Photoluminescence Quenching and Exciton Lifetimes

**Fig. 7:**
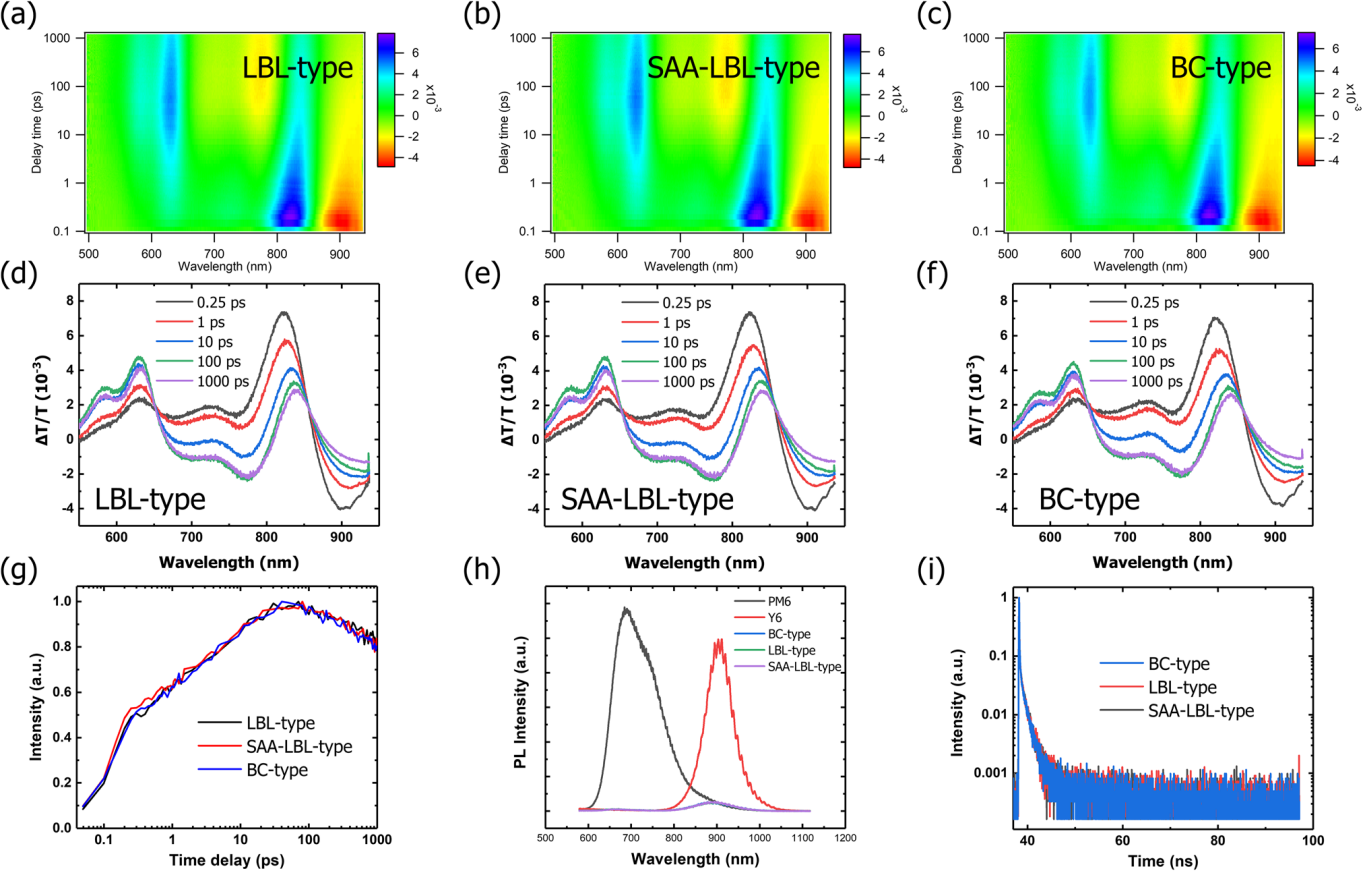
Color plots of the TA spectra for **a** LBL-type, **b** SAA-LBL-type and **c** BC-type blend films in the range of 500–930 nm. TA spectra of **d** LBL-type, **e** SAA-LBL-type and **f** BC-type blend films in the range of 500–960 nm of the blend films at different delay times. **j** TA traces of three blend films probed at 630 nm. **h** PL spectra of pristine PM6 and Y6 films, BC-type, LBL-type, and SAA-LBL-type blend films. **i** TRPL spectra of BC-type, LBL-type and SAA-LBL-type blend films

To investigate the effect of SAA-LBL-type morphology on charge generation process and explain higher *J*_sc_ of SAA-LBL-type devices, time-resolved absorption measurements are carried out. 800 nm is used as excitation wavelength to selectively excite the acceptors in D:A blend films. The results shown in Fig. 7a–f display the photo-induced bleaching (PB) of three structures. After excitation, the PB signals of Y6 located at 810 nm gradually descends, meanwhile, increased PB signals of PM6 at 630 nm could be observed. This clearly shows hole transfer from acceptor to donor. Among them, SAA-LBL-type has strongest intensity in 630 nm which means effective hole transfer, while BC-type has the weakest one. Figure S14 presents the TA traces of BC-type, LBL-type and SAA-LBL-type films at different wavelengths. Furthermore, the dynamic behaviors of hole transfer are researched by analyzing the PB signals at 630 nm versus time delay (Fig. 7g). Fitted with biexponential function, the charge transfer lifetimes could be calculated as follows. In LBL-type film, ${\tau }_{1}$ = 0.22 ps and ${\tau }_{2}$ = 6.88 ps; in SAA-LBL-type film, ${\tau }_{1}$ = 0.10 ps and ${\tau }_{2}$ = 5.02 ps; in BC-type, ${\tau }_{1}$ = 0.15 ps and ${\tau }_{2}$ = 6.05 ps, where ${\tau }_{1}$ is usually referred to the dissociation of the acceptor exciton formed at the D:A interface, while ${\tau }_{2}$ relates to diffusion-limited dissociation of bulk excitons [43–45]. It is plausible that BC-type and SAA-LBL-type structure possess slightly faster hole transfer than LBL-type. Moreover, SAA-LBL-type exhibits smallest ${\tau }_{2}$, which could be explained by that appropriate domain size and Y6 diffusion could shorten the exciton diffusion length thus, exciton transfers to interface of D and A faster and reduces the deexcitation and recombination in the process of diffusion. However, because of the meandering route of exciton diffusion and island-like zone in BC-type structure, it costs longer time for exciton diffusion. LBL-type, resulted from partly limited Y6 diffusion length and D:A interfacial area, has the longest distance of exciton diffusion to some extent.

Photoluminescence (PL) spectra could study the charge transfer from one phase to another. The PL results of pristine PM6 and Y6 films as well as three blend films processed with different methods are shown in Fig. 7h. It is perceptible that PM6 and Y6 films exhibit distinct PL peaks but all blend films show negligible PL intensities. Further calculation indicates the PL quenching efficiency exceeds 90%, suggesting that no matter BC-type, LBL-type and SAA-LBL-type morphology have ample D:A interfacial area for charge transfer. Time-resolved Photoluminescence (TRPL) is performed to investigate the average fluorescence lifetime ($\tau$). It shows in Fig. 7i that BC-type structure has the smallest $\tau$ with 1.12 ns, while LBL-type and SAA-LBL-type are estimated as 1.13 and 1.16 ns. These results indicate that all three structures have similar exciton lifetimes, which is not difficult to understand as the active materials we adopted to fabricate devices are the same after all. In other words, the increased *J*_sc_ and FF are originated from the optimized morphology.

### Universality of SAA-LBL

Since the SAA-LBL-type structure enables great successfulness in PM6:Y6 devices, it is applied to high efficiency PM6:L8-BO blend to verify its generality (Table 1). Excitingly, the devices based on PM6:L8-BO exhibit a PCE of 19.02% with SAA-LBL-type structure, higher than that of LBL-type (18.72%) and BC-type (18.56%). Besides, the various FAs with different polarities could satisfy the requirement of various D:A types for diverse suitable phase separation. Inspired by the advantage of SAA-LBL method, the universality of this strategy to thick film fabrication is also explored. Cai et al. [46] found the excitons are mainly generated near PEDOT:PSS for thick film since the light incidence direction is from PEDOT:PSS side. Hence, the effective exciton dissociation and charge transfer are the key elements for the whole photo-to-electron process. Exactly, our method could help construct more even vertical phase distribution, thus relieving the restrictive factors that make it hard to improve the efficiency of thick film devices. Therefore, 250 nm-thick film device based on PM6:L8-BO achieves a PCE of 16.44% with the aid of FA-C12 (Fig. S15), and this is one of the highest PCE records for thick film binary OSCs reported so far. Benefited from the augmented absorption of thick film, the *J*_sc_ reaches a record value of 27.30 mA/cm^2^ among PM6:L8-BO based devices. The serious decrease of FF caused by low mobility of charges may be responsible for the falling PCE of thick film devices.

## Conclusions

In summary, we have developed a solid additive-assisted layer-by-layer processing to fine-tune the morphology and optimize the device performance of organic solar cells. By modifying the miscibility of PM6 and the solid additives, i.e., the fatty acids (FAs), the appropriate phase separation is induced with tridecanoic acid being solid additive mixed with precursor donor solution, and this facilitates the inter-diffusion between donor and acceptor and induces a more ideal vertical phase separation, leading to better balance of charge generation, transport and collection. Consequently, the PCE of OSCs based on PM6:Y6 increases from 17.52% (LBL-type) to 18.16% (SAA-LBL-type) with the simultaneous enhancement of *J*_sc_ of 27.74 mA cm^−2^ and FF of 76.7%. The achieved champion efficiency is also one of the highest among PM6:Y6-based binary OSCs. Moreover, the generality of SAA-LBL processing is confirmed by applying it to more diverse OSC systems. Particularly, with SAA-LBL, the OSCs based on PM6:L8-BO reach high PCEs of 19.02% and 16.44%, which are the best results among binary OSCs with optimal and thick films, respectively. Our work demonstrates a simple but efficacious method to fabricate high-performance OSCs for future commercialization.

### Supplementary Information

Below is the link to the electronic supplementary material.

Supplementary file1 (PDF 1631 KB)
