# Supplementary material for: Solid Additive-Assisted Layer-by-Layer Processing for 19% Efficiency Binary Organic Solar Cells
Source: Nanomicro Lett. 2023 Apr 10;15:92. doi: 10.1007/s40820-023-01057-x (PMC10086087; doi:10.1007/s40820-023-01057-x)
Supplement: Supplementary file 1 — Supplementary file1 (PDF 1631 KB) [file 40820_2023_1057_MOESM1_ESM.pdf]

Supporting Information for

Solid Additive Assisted Layer-by-Layer Processing for 19% Efficiency Binary Organic Solar Cells

Guanyu Ding[1, #], Tianyi Chen[1, #], Mengting Wang[1], Xinxin Xia[2], Chengliang He[1], Xiangjun Zheng[1], Yaokai Li[1], Di Zhou[4], Xinhui Lu[2], Lijian Zuo[1, 3, *], Zhikang Xu[4, 5], Hongzheng Chen[1, 5, *]

[1]State Key Laboratory of Silicon Materials, Department of Polymer Science and Engineering, Zhejiang University, Hangzhou 310027, P. R. China

[2]Department of Physics, The Chinese University of Hong Kong, New Territories, Hong Kong, 999077, P. R. China

[3]Zhejiang University-Hangzhou Global Scientific and Technological Innovation Center, Hangzhou 310014, P. R. China.

[4]MOE Key Laboratory of Macromolecular Synthesis and Functionalization, Department of Polymer Science and Engineering, Zhejiang University, Hangzhou 310027, P. R. China

[5]Key Lab of Adsorption and Separation Materials & Technologies of Zhejiang Province, Zhejiang University, Hangzhou 310027, P. R. China

[#]Guanyu Ding and Tianyi Chen contributed equally to this work.

*Corresponding authors. E-mail: zjuzlj@zju.edu.cn (Lijian Zuo); hzchen@zju.edu.cn (Hongzheng Chen)

S1 Materials and Methods

Instrument. UV-vis absorption spectra were recorded on a U-4100 (HITACHI) UV-vis spectrophotometer. Topographic images of the films were obtained on a VeecoMultiMode atomic force microscopy (AFM) in the tapping mode using an etched silicon cantilever at a nominal load of ~2 nN, and the scanning rate for a 10 μm × 10 μm image size was 1.5 Hz and for a 1 μm × 1 μm image size was 1.0 Hz.

Materials. PM6, Y6 and PDINN were purchased from Solarmer Inc. L8-BO was purchased from Chasing Light Technology Company Limited. PEDOT:PSS was purchased from Heraeus Company Limited. Fatty acids (FAs) with different carbon

chain lengths were purchased from Aladdin Inc. The fusion temperature of FA-C9, FA-C12, and FA-C16 is 304 K, 315 K, and 333 K, respectively, and their boiling points are 422 K, 509 K, and 500 K, respectively. All reagents and solvents were purchased from commercial sources and were used without further purification.

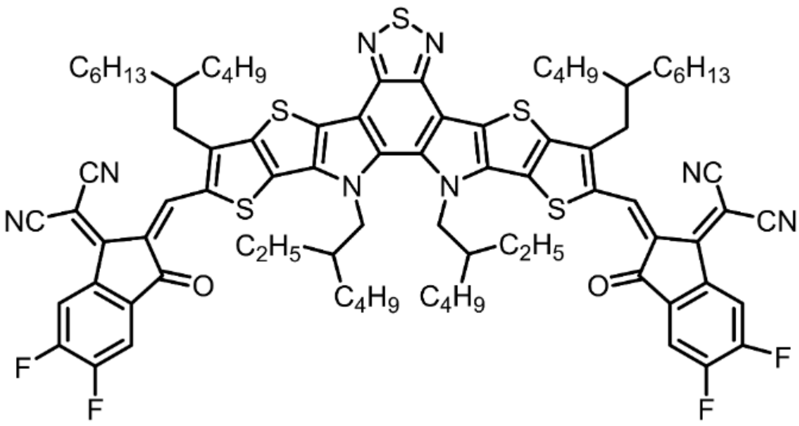

L8-BO

Equation for J_{sc} to P_{light}.

$$J_{SC} \propto P_{light}^{\alpha} \tag{S1}$$

Charge Carrier Mobility Measurements. The charge carrier mobilities of blend films were measured using the space charge limited current (SCLC) method. Hole-only devices were fabricated in a structure of ITO/PEDOT:PSS/Active Layer/MoO_3/Ag, electron-only devices were fabricated in a structure of ITO/ZnO/Active Layer/PDINN/Ag. The device characteristics were extracted by modeling the dark current under forward bias using the SCLC expression described by the Mott-Gurney law [1]:

$$J = \frac{9}{8}\varepsilon_r\varepsilon_0\mu\frac{V^2}{L^3} \tag{S2}$$

Here, $\varepsilon_r \approx 3$ is the average dielectric constant of the blend film, ε_0 is the permittivity of the free space, μ is the carrier mobility, L is the thickness of the film, and V is the applied voltage.

Exciton Dissociation Probability (P_{diss}) and Charge Collection Probability (P_{coll}) Calculation. The current density under illumination (J_L) and in the dark (J_D) with the

change of voltage are investigated. The photocurrent density (J_{ph}) could be calculated by the equation of

$$J_{ph} = J_L - J_D \qquad \text{(S3)}$$

The voltage in which the generated J_{ph} is zero is called bias voltage (V_{bi}), and the effective voltage (V_{eff}) is defined as

$$V_{eff} = V_{bi} - V \qquad \text{(S4)}$$

where V is the changeable applied voltage. P_{diss} is decided by the equation

$$P_{diss} = J_{sc} \,/\, J_{sat} \qquad \text{(S5)}$$

where J_{sat} is the saturate photocurrent density at high V_{eff}. Similarly, P_{coll} could be estimated by the equation

$$P_{diss} = J_{max} \,/\, J_{sat} \qquad \text{(S6)}$$

where J_{max} is the current density when device reaches maximum output power [2].

Time-of-Flight Secondary Ion Mass Spectrometry (TOF-SIMS) Measurements. Cs^+ was used as the sputter source with a 500 eV energy and 40 nA current. The typical sputter area was 200 µm by 200 µm.

Surface Tension Measurements. Contact angle measurements were performed using water and diiodomethane by sessile drop analysis.

The Flory-Huggins Interaction Parameter Calculation.

$$\chi^{D-Add} = \left(\sqrt{\gamma^{D}} - \sqrt{\gamma^{Add}}\right)^2 \qquad \text{(S7)}$$

GISAXS/GIWAXS measurements. GISAXS/GIWAXS measurements were carried out with a Xeuss 2.0 SAXS/WAXS laboratory beamline using a Cu X-ray source (8.05 keV, 1.54 Å) and a Pilatus3R 300K detector. The incidence angle is 0.2°. The thin film samples (including pure and blend films) were prepared on Si wafer substrates by spin coating the precursor solutions.

S2 Supplementary Figures and Tables

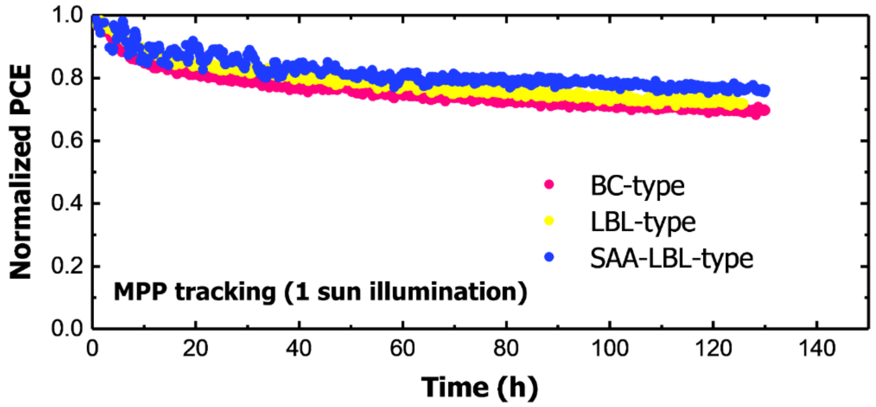

Fig. S1 Photostability of the BC-type, LBL-type and SAA-LBL-type devices with MPP tracking under 1 sun illumination

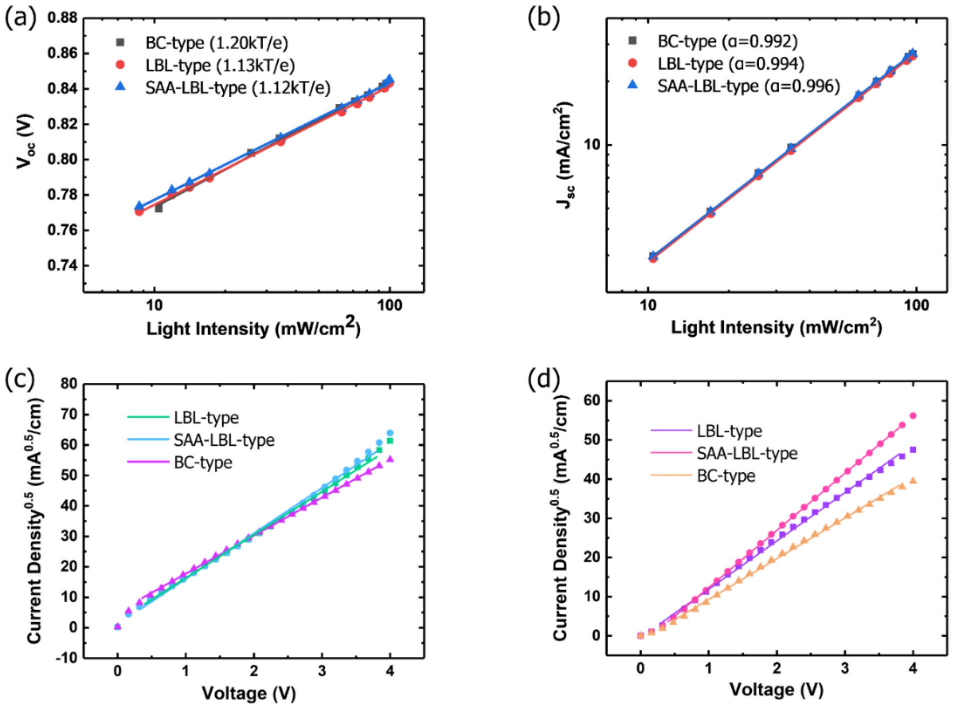

Fig. S2 The dependence of **a** V_{oc} and **b** J_{sc} on light intensity (P_{light}) of BC-type, LBL-type and SAA-LBL-type devices. SCLC curves of **c** hole-only and **d** electron-only BC-type, LBL-type and SAA-LBL-type devices

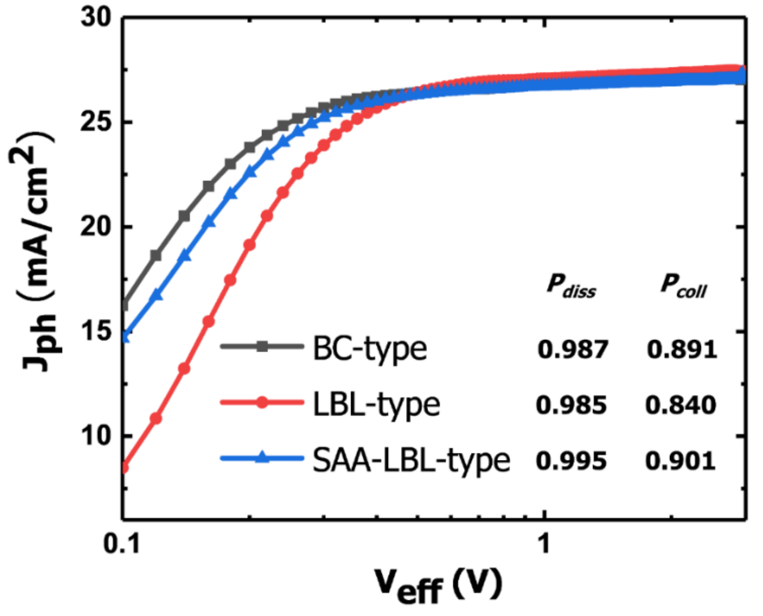

Fig. S3 J_{ph}-V_{eff} curves for BC-type, LBL-type and SAA-LBL-type devices

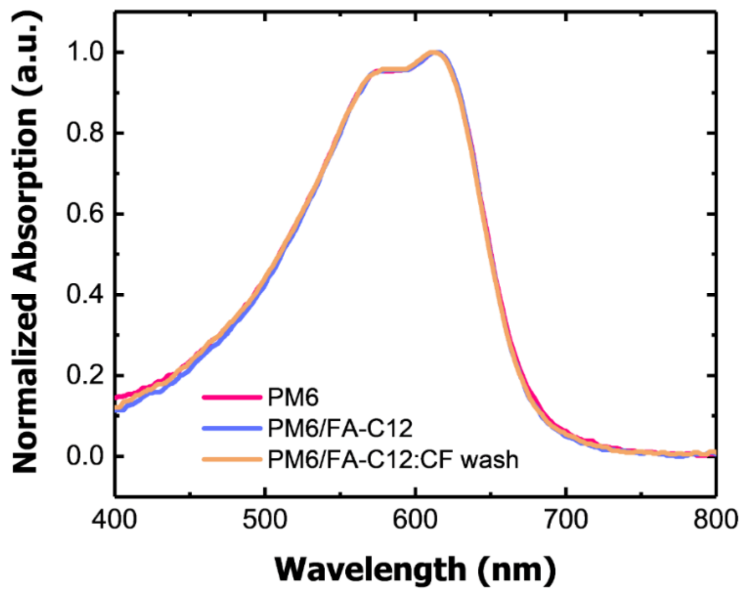

Fig. S4 UV-Vis spectra of pristine PM6 film, PM6/FA-C12 blend film and blend film after chloroform wash

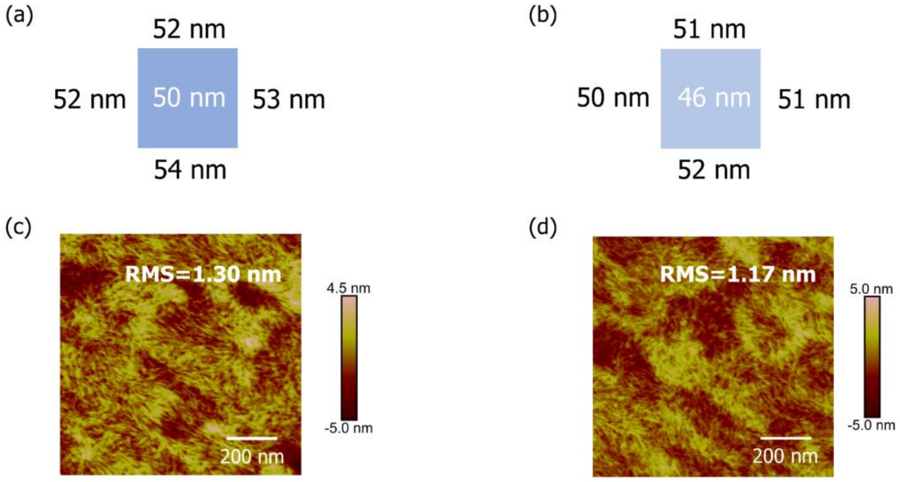

Fig. S5 a, b Thickness of different positions for PM6/FA-C12 film before and after chloroform washing. AFM height images of PM6/FA-C12 film **c** before and **d** after chloroform washing

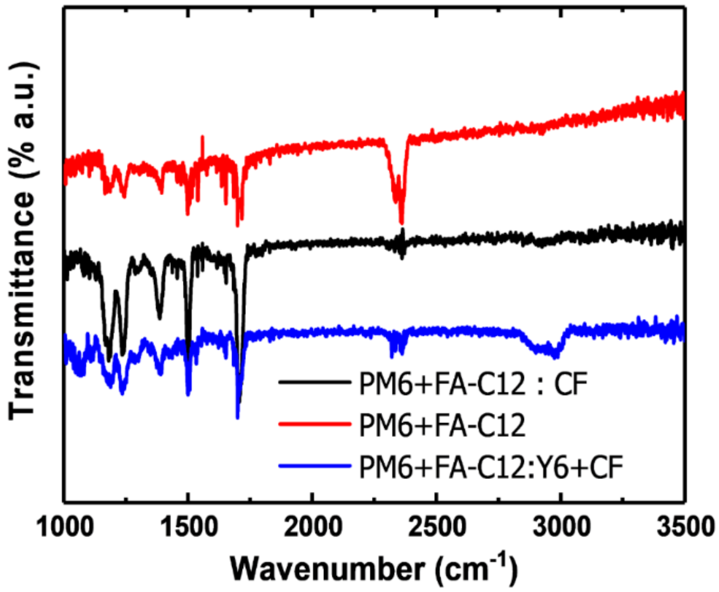

Fig. S6 ATR-FTIR spectra of PM6 films treated with different operations

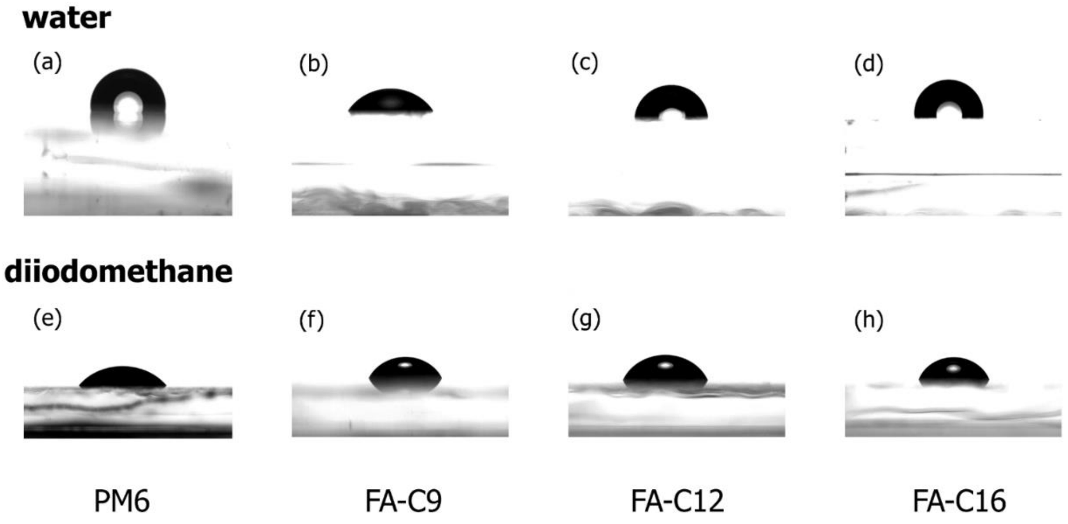

Fig. S7 Contact angle images of pristine **a, e** PM6, **b, f** FA-C9, **c, g** FA-C12, and **d, h** FA-C16 films with water and diiodomethane droplet on top

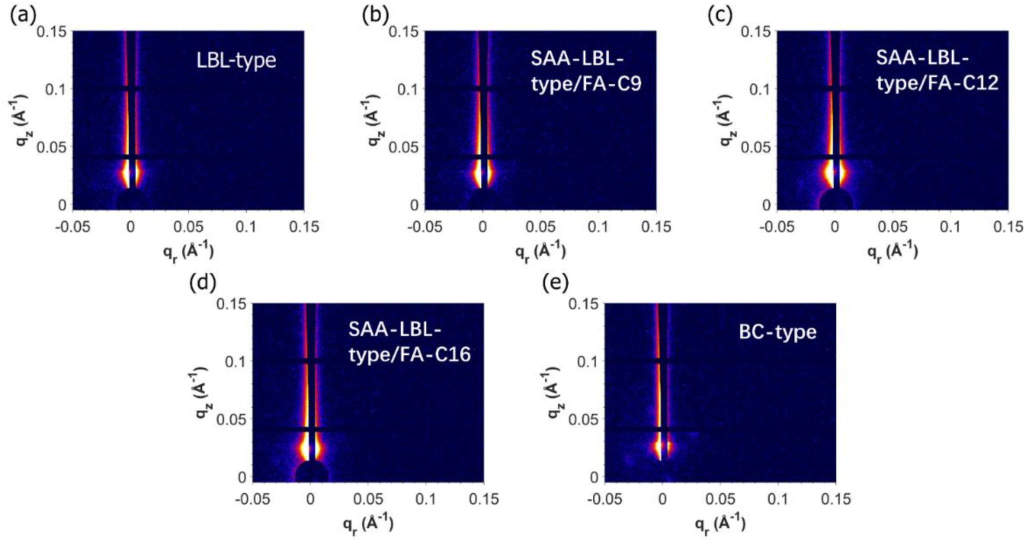

Fig. S8 GISAXS images of **a** LBL-type, **b** SAA-LBL-type/FA-C9, **c** SAA-LBL-type/FA-C12, **d** SAA-LBL-type/FA-C16 and **e** BC-type blend films

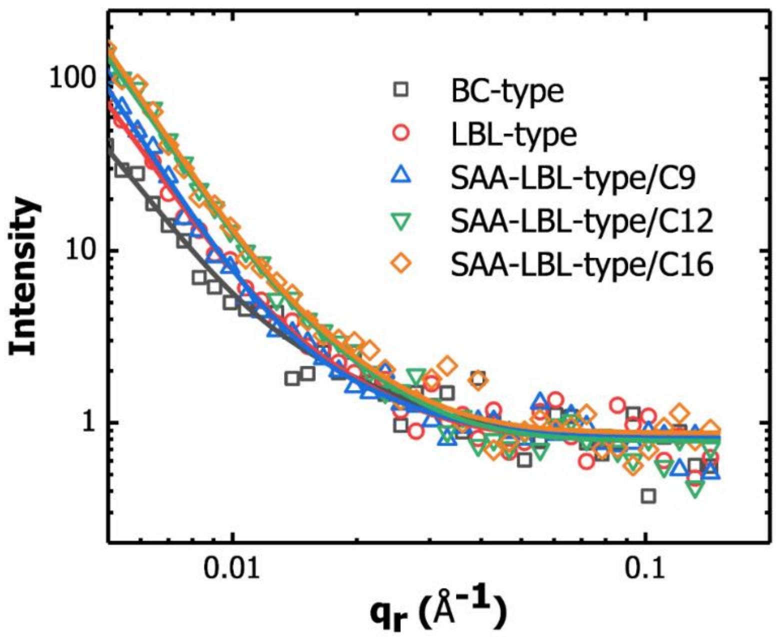

Fig. S9 Linecut and fitting curves for GISAXS intensity of the corresponding films along the q_r axis

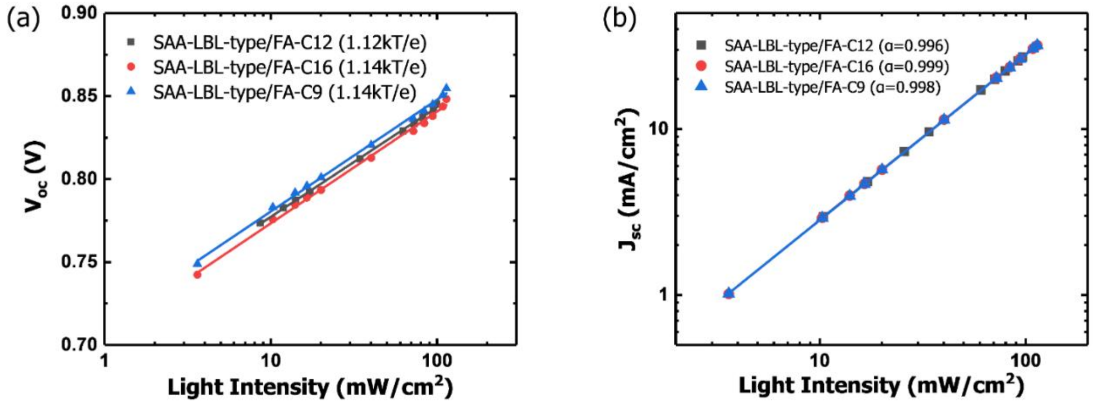

Fig. S10 The dependence of **a** V_{oc} and **b** J_{sc} on light intensity (P_{light}) of SAA-LBL-type devices regulated by FA-C12, FA-C16 and FA-C9

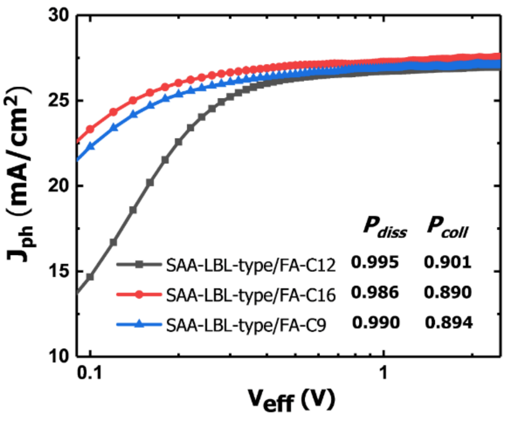

Fig. S11 J_{ph}-V_{eff} curves for SAA-LBL-type devices regulated by FA-C12, FA-C16 and FA-C9

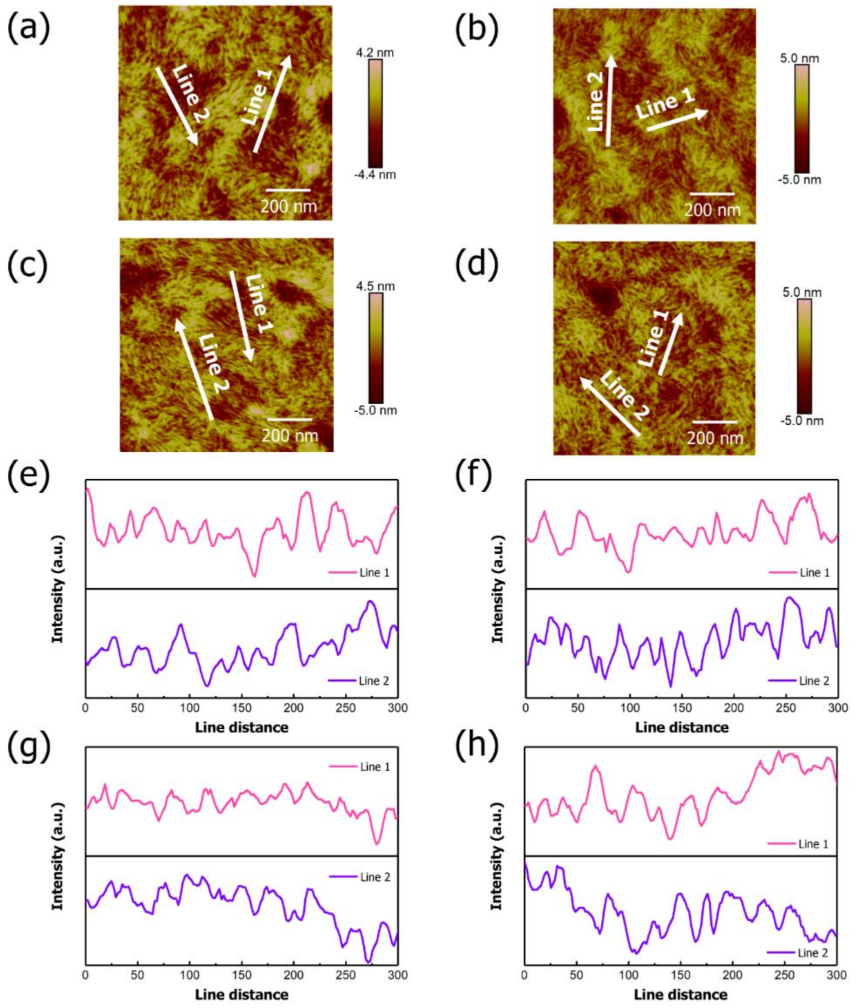

Fig. S12 AFM height images of **a** pristine PM6 film, **b** PM6 film with 5% FA-C12, **c** PM6 film with 10% FA-C12, and **d** PM6 film with 15% FA-C12. Line profiles of 1 μm × 1 μm AFM signals of **e** pristine PM6 film, **f** PM6 film with 5% FA-C12, **g** PM6 film with 10% FA-C12, **h** PM6 film with 15% FA-C12

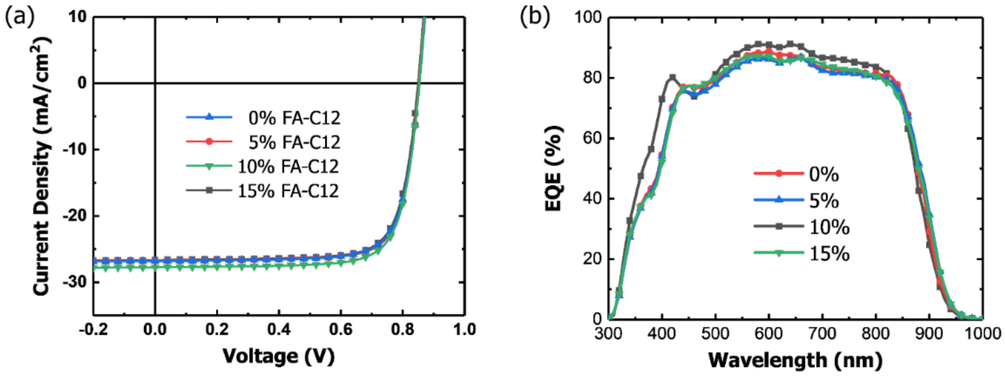

Fig. S13 a *J-V* and **b** EQE curves of SAA-LBL-type devices with different ratios of FA-C12

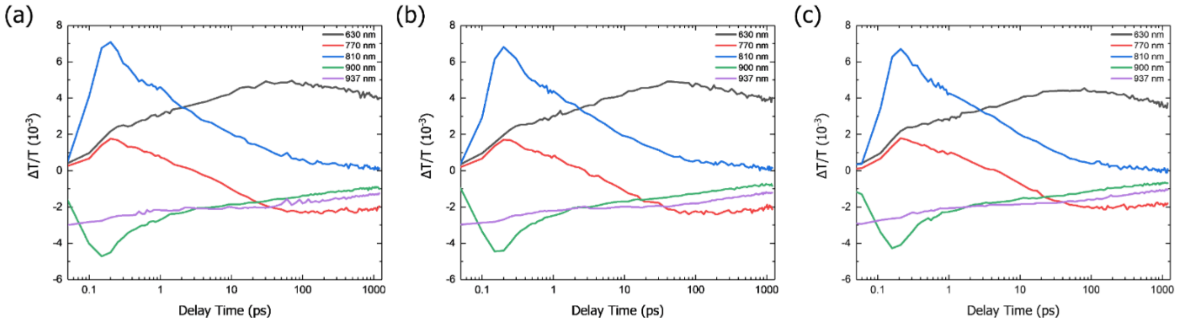

Fig. S14 TA traces of **a** LBL-type, **b** SAA-LBL-type, and **c** BC-type blend films probed at different wavelengths

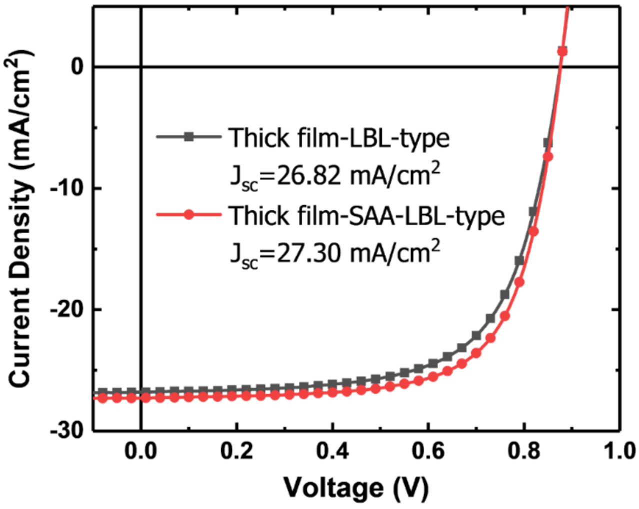

Fig. S15 *J-V* curves of LBL-type and SAA- LBL-type devices based on PM6:L8BO

Table S1 Comparison of device parameters between this work and references

J_{sc} (mA/cm^2)	PCE (%)	Ref.
27.74	18.16	Text
27.01	17.44	[3]
26.42	16.22	[4]
27.68	17.18	[5]
26.49	17.02	[6]
26.78	17.9	[7]
27.99	17.01	[8]
26.6	17.2	[9]
27.1	17.6	[10]
26.5	17.36	[11]
26.88	17.4	[12]
26.52	16.53	[13]
26.95	17.73	[14]
27.46	18.01	[15]
26.15	17.48	[16]
26.43	17.59	[17]
26.4	17.6	[18]
25.2	16.8	[19]
27.0	17.6	[20]

Table S2 Photovoltaic parameters of the BC-type devices based on PM6:Y6 blends with 10% (w/w) FAs

Solid additive	V_{oc} (V)	J_{sc} (mA/cm^2)	FF (%)	PCE (%)
/	0.85<br>(0.85±0.002)	26.66<br>(26.69±0.15)	74.1 (74.2±0.17)	16.80<br>(16.87±0.13)
FA-C5	0.85<br>(0.85±0.002)	26.54<br>(26.38±0.13)	73.7 (73.5±0.16)	16.65<br>(16.53±0.14)
FA-C12	0.85<br>(0.85±0.002)	26.27<br>(26.11±0.18)	76.0 (75.9±0.12)	16.89<br>(16.69±0.17)
FA-C16	0.85<br>(0.85±0.002)	26.05<br>(25.87±0.20)	75.2 (73.5±0.15)	16.59<br>(16.41±0.19)

Table S3 Summary of contact angles (θ), surface tensions (γ), and Flory–Huggins interaction parameters (χ) for PM6, FA-C9, FA-C12, and FA-C16 films

Surface	θ_{water} (°)	$\theta_{diiodomethane}$ (°)	γ (mN/m)	$\chi^{D\text{-}Add}$
PM6	99.4	52.1	34.9	
FA-C9	71.8	61.9	34.6	0.0073
FA-C12	85.2	65.7	27.5	0.31
FA-C16	104.9	68.5	23.9	0.82

Table S4 Crystal coherence lengths of the (100) peak and the d-spacing for pristine PM6, PM6/FA-C12 and pristine Y6 films

Active Layer	Location /Å^{-1}	d-spacing /Å	FWHM/Å^{-1}	CCL/Å
PM6	0.28	22.4	0.148	38.2
PM6/FA-C12	0.28	22.4	0.160	35.3
Y6	0.31	20.3	0.259	21.8

Table S5 Crystal coherence lengths of the (010) peak and the d-spacing for pristine PM6, PM6/FA-C12 and pristine Y6 films

Active Layer	Location /Å^{-1}	d-spacing /Å	FWHM/Å^{-1}	CCL/Å
PM6	1.69	22.4	0.353	16.0
PM6/FA-C12	/	/	/	/
Y6	1.81	3.47	0.385	14.7

Table S6 Crystal coherence lengths of the (100) peak and the d-spacing for BC-type, LBL-type and SAA-LBL-type films

Active Layer	Location /Å^{-1}	d-spacing /Å	FWHM/Å^{-1}	CCL/Å
BC-type	0.29	21.7	0.199	28.4
LBL-type	0.28	22.4	0.180	31.4
SAA-LBL-type	0.29	22.0	0.163	34.7

Table S7 Crystal coherence lengths of the (010) peak and the d-spacing for BC-type, LBL-type and SAA-LBL-type films

Active Layer	Location /Å^{-1}	d-spacing /Å	FWHM/Å^{-1}	CCL/Å
BC-type	1.73	3.63	0.485	11.7
LBL-type	1.74	3.61	0.492	11.5
SAA-LBL-type	1.76	3.57	0.543	10.4

Supplementary References

[S1] A.L. Philip. Electronic processes in ionic crystals (mott, n. F.; gurney, r. W.). J. Chem. Educ. (1941). https://doi.org/10.1021/ed018p249.1

[S2] A.K.K. Kyaw, D.H. Wang, D. Wynands, J. Zhang, T.Q. Nguyen et al., Improved light harvesting and improved efficiency by insertion of an optical spacer (ZnO) in solution-processed small-molecule solar cells. Nano Lett. **13**(8), 3796-3801 (2013). https://doi.org/10.1021/nl401758g

[S3] X. Xiong, X. N. Xue, M. Zhang, T. Y. Hao, Z. Y. Han et al., Melamine-doped cathode interlayer enables high-efficiency organic solar cells. ACS Energy Lett. **6**(10), 3582-3589 (2021). https://doi.org/10.1021/acsenergylett.1c01730

[S4] B. Chen, Q. Wang, B. B. Wang, W. T. Miao, G. P. Zhang et al., P-nitrophenol-terminated alkyl side chain substituted polymer as high dielectric constant polymer additive enables efficient organic solar cells. Opt. Mater. **127**, 112347 (2022). https://doi.org/10.1016/j.optmat.2022.112347

[S5] S. S. Chen, C. L. He, Y. K. Li, T. Y. Chen, X. X. Xia112347 Improving the device performance of organic solar cells with immiscible solid additives. J. Mater. Chem. C **10**(7), 2749-2756 (2022). https://doi.org/10.1039/d1tc04222j

[S6] Z. J. Fu, W. Y. Yu, H. Song, G. Q. Zhang, H. Y. Chen et al., A new simple volatile solid additive triggers morphological optimization and performance stabilization in polymer solar cells. Sustain. Energy Fuels **6**(9), 2191-2197 (2022). https://doi.org/10.1039/d2se00199c

[S7] L. Z. Guo, Q. D. Li, J. X. Ren, Y. J. Xu, J. B. Zhang et al., Halogenated thiophenes serve as solvent additives in mediating morphology and achieving efficient organic solar cells. Energy Environ. Sci. (2022). https://doi.org/10.1039/d2ee02553a

[S8] K. E. Hung, Y. S. Lin, Y. J. Xue, H. R. Yang, Y. Y. Lai et al., Non-volatile perfluorophenyl-based additive for enhanced efficiency and thermal stability of nonfullerene organic solar cells via supramolecular fluorinated interactions. Adv. Energy Mater. **12**(12), (2022). https://doi.org/10.1002/aenm.202103702

[S9] D. H. Lee, D. H. Kim, T. Kim, D. C. Lee, S. Cho et al., Solid-solvent hybrid additive for the simultaneous control of the macro- and micro-morphology in non-fullerene-based organic solar cells. Nano Energy **93**, (2022). https://doi.org/10.1016/j.nanoen.2021.106878

[S10] H. Lu, K. Chen, R. S. Bobba, J. Shi, M. Li et al., Simultaneously enhancing exciton/charge transport in organic solar cells by an organoboron additive. Adv. Mater. **34**(42), e2205926 (2022). https://doi.org/10.1002/adma.202205926

[S11] H. Mao, L. Zhang, L. Wen, L. Huang, L. Tan et al., Nanoimprint lithography-dependent vertical composition gradient in pseudo-planar heterojunction organic solar cells combined with sequential deposition. Adv. Funct. Mater. 2209152 (2022). https://doi.org/10.1002/adfm.202209152

[S12] H. Song, D. Hu, J. Lv, S. Lu, C. Haiyan et al., Hybrid cathode interlayer enables 17.4% efficiency binary organic solar cells. Adv. Sci. **9**(8), e2105575 (2022). https://doi.org/10.1002/advs.202105575

[S13] Y. Su, L. Zhang, Z. Ding, Y. Zhang, Y. Wu et al., Carrier generation engineering toward 18% efficiency organic solar cells by controlling film microstructure.

Adv. Energy Mater. **12**(19), 2103940 (2022). https://doi.org/10.1002/aenm.202103940

[S14] Y.-N. Yang, X.-M. Li, S.-J. Wang, X.-P. Duan, Y.-H. Cai et al., An organic small molecule as a solid additive in non-fullerene organic solar cells with improved efficiency and operational stability. Chin. J. Polym. Sci. (2022). https://doi.org/10.1007/s10118-022-2860-8

[S15] K. Yu, W. Song, J. Ge, K. Zheng, L. Xie et al., 18.01% efficiency organic solar cell and 2.53% light utilization efficiency semitransparent organic solar cell enabled by optimizing pm6:Y6 active layer morphology. Sci China Chem. **65**(8), 1615-1622 (2022). https://doi.org/10.1007/s11426-022-1270-5

[S16] L. Zhang, H. Mao, L. Huang, L. Hu, X. Wang et al., Achieving improved stability and minimal non-radiative recombination loss for over 18% binary organic photovoltaics via versatile interfacial regulation strategy. Sci. China Chem. **65**(8), 1623-1633 (2022). https://doi.org/10.1007/s11426-022-1300-1

[S17] X. J. Zhou, X. Li, Y. J. Yan, F. H. Zhang, J. Zhou et al., Improved current density and fill factor of non-fullerene organic solar cells prepared under solvent vapor atmosphere. Solar Rrl. **6**(9), 2200424 (2022). https://doi.org/10.1002/solr.202200424

[S18] M. Zi, X. Chen, S. Tan, C. Weng, B. Zhao, Organic solar cells with efficiency of 17.6% and fill factor of 78.3% based on perylene-diimide derivative as cathode interface layer. Chem. Eng. J. **443,** 136455 (2022). https://doi.org/10.1016/j.cej.2022.136455

[S19] R. Geng, P. Liu, R. Pan, H. Xu, S. Gao et al., Self-assembled monolayers featuring multi-chlorinated carbazole unit for improving hole extraction efficiency in organic photoelectronic device. Chem. Eng. J. **454**, 140138 (2023). https://doi.org/10.1016/j.cej.2022.140138

[S20] X. Liao, Q. Li, J. Ye, Z. Li, J. Ren et al., Solid-liquid convertible fluorinated terthiophene as additives in mediating morphology and performance of organic solar cells. Chem. Eng. J. **453**, 139489 (2023). https://doi.org/10.1016/j.cej.2022.139489
